# 

**Published:** 1908-04-11

**Authors:** 


### A Memorial to a Judge.

### THE CASE OF "CROPPER *v.* STOKES."

The following is an account published in *The Times* of April 7 of the presentation of a Memorial to Judge Edge, with reference to observations recently made by His Honour concerning Dr. F. H. Stokes' evidence in the case of "Cropper *v.* Stokes."

"At Clerkenwell County Court on April 6, an application was made to Judge Edge in the case of "Cropper *v.* Stokes," which recently came before the Court. Miss Alice Cropper, of Horsham, sued Dr. Henry Fraser Stokes, of Highbury Place, Canonbury, for the return of £17. In the evidence it was stated that Dr. Stokes, who attended the plaintiff's late father, promised that if a cheque was given to him for £42 he would return £17, that being the difference between a charge of £5 5s. per visit and £3 3s. per visit. Several applications were made, and the defendant was written to, but the money was never forthcoming. Dr. Stokes, in evidence, denied that he ever consented to charge £3 3s. instead of £5 5s., and said that the plaintiff's brother called upon him in April or May and said he could make the charge £10 10s. per visit, because it would keep down the estate duty. This Mr. Cropper denied. In giving his decision Judge Edge said he did not believe one particle of Dr. Stokes's evidence. He found for the plaintiff for the amount claimed, and refused leave to appeal.

"Mr. H. F. Dickens (instructed by Messrs. Hempsons, solicitors to the Medical Defence Union) read a memorial to Judge Edge, in which the signatories said they were deeply concerned to learn that in giving judgment his Honour did not believe the evidence given by Dr. Stokes, inferentially imputing perjury to him. They did not presume to discuss the merits of the case or to question the judgment; but it was quite incredible to them that Dr. Stokes could have permitted perjury. Later the memorial urged that any inaccuracy in Dr. Stokes's account must have been the result of misunderstanding as to what passed between himself and the plaintiff's witnesses, and could not have been intentionally untrue, and that his omission to answer the letter could not have been due to any wrongful intention. The memorial was signed, among others, by Sir Douglas Powell, Sir Thomas Barlow, Sir Victor Horsley, and other medical men, besides clergymen and others.

"Judge Edge said that in the course of his experience he had never heard of a proceeding of the kind before—that was, the presenting of a memorial to a Judge who in the course of his judicial duties thought it his duty to make observations on the evidence given by a witness before him. In that case he was glad to have that memorial, because if he had unintentionally done a wrong, and certainly without any feeling against Dr. Stokes in the least except such as was caused by the manner in which he gave his evidence, then he was glad to be set right. In the present case all the parties concerned were equally unknown to him, and his observations were made solely on the evidence before him, and were made because he considered it his imperative duty to make them. Dr. Stokes, it appeared, was and had been long known to many learned and distinguished men, and they believed him incapable of stating that which he knew to be untrue, and that the evidence given before him upon which he commented so strongly was given under misapprehension and confused recollection, but believed by him to be true. There were circumstances in the evidence given by him which upon reflection led him (the Judge) to think that such might have been the case. He noted at the time, and commented upon it in his judgment, that the evidence given by Dr. Stokes did not tally with the instructions given to his learned counsel. If his memory failed him as to the instructions given to his solicitor, though some considerable time before the trial, how much more might his recollection of circumstances which had occurred long before that have been likely to have become confused and blurred? The gentlemen who had signed the memorial thought that in the stress of carrying on a large practice that was a solution of his apparent untruthfulness, and he was quite willing to accept that explanation."

